# Effects of Chronic Binge Alcohol Consumption on Alzheimer’s Disease Neuropathogenesis in 3xTg‐AD mice

**DOI:** 10.1002/alz.092570

**Published:** 2025-01-03

**Authors:** Lucy J. Sloan, Paula M. Chilton, Aakarsha V. Rao, JingWen Zhang, Scott A. Myers, Sreelatha Reddy, Jiyeon Lee, Walter Rodriguez, Leila Gobejishvili, Julia Chariker, Eric Rouchka, Smita S. Ghare, Craig J. McClain, Shirish Barve

**Affiliations:** ^1^ University of Louisville, Louisville, KY USA; ^2^ Norton Neuroscience Institute, Louisville, KY USA; ^3^ University of Louisville, Lousiville, KY USA; ^4^ Norton Neuroscience Institute, Lousiville, KY USA

## Abstract

**Background:**

Chronic, excessive alcohol consumption causes neurodegeneration and is associated with an increased risk for Alzheimer’s disease (AD) and other dementias. Moreover, there has been a consistent rise in alcohol consumption in older adults in the past few decades. However, there is minimal research showing how alcohol consumption affects AD neuropathogenesis and biological mechanisms remain unclear. We hypothesize that mid‐life chronic binge alcohol consumption accelerates AD neuropathology in the hippocampus, among the first brain regions affected by AD, through dysregulation of processes associated with AD pathogenesis.

**Methods:**

Female triple transgenic (3xTg; n = 15) mice and non‐transgenic (NonTg) control mice (B6.129; n = 13) were given EtOH binges twice/week (4g/kg) by oral gavage for 3.5 months (from 6‐9.5 months) while untreated controls (3xTg n = 6 and NonTg n = 4) received sham gavages. Memory was evaluated with the novel object recognition test (NORT). RNA was extracted from the mid‐brain region of one hemisphere for RNA‐Sequencing (Illumina NextSeq2000; STAR/HTSeq/DESeq2 pipeline) and qPCR, while protein lysates from the opposite hemisphere’s midbrain region was used for Western blot (WB) analyses. Plasma neurofilament light chain (NfL) was measured with a MesoScale Discovery kit.

**Results:**

One week after the final EtOH gavage, hippocampal‐dependent memory was significantly impaired in EtOH binged 3xTg mice, while NonTg mice were unaffected. Similarly, EtOH binge slightly increased plasma NfL in 3xTg mice, indicating persistent EtOH‐induced neurodegeneration, while nonTg mice showed a significant decrease. RNA‐seq from the mid‐brain region containing the hippocampus revealed that chronic EtOH binge significantly affected 8477 genes (4473 up‐ and 4004 down‐regulated) in 3xTg mice, while only 4 genes were significantly affected (2 up‐ and 2 down‐regulated) in nonTg mice. Enrichment analyses showed EtOH altered several processes associated with neurodegeneration in 3xTg mice including regulation of synaptic plasticity and autophagy.

**Conclusions:**

Results indicate that 3xTg mice are more sensitive to EtOH‐mediated effects on neurodegeneration and memory impairment than nonTg controls. There was also significant EtOH‐induced dysregulation of AD‐related processes in the mid‐brain of 3xTg mice, a region affected early by AD neuropathology. Together, these data show that chronic mid‐life EtOH binging accelerates early AD neuropathology in 3xTg mice.

